# Methods of Suicide Among Nurses Globally: Examination of Epidemiological and Cohort Evidence

**DOI:** 10.1177/10783903251326840

**Published:** 2025-03-20

**Authors:** Elizabeth Kreuze, Elizabeth I. Merwin, Janet York

**Affiliations:** 1 Elizabeth Kreuze, PhD, RN, ATC, College of Nursing and Health Innovation, University of Texas at Arlington, Arlington, TX, USA; 2 Elizabeth I. Merwin, PhD, RN, FAAN, College of Nursing and Health Innovation, University of Texas at Arlington, Arlington, TX, USA; 3 Janet York, PhD, PMHCS-BC, FAAN, College of Nursing, Medical University of South Carolina, Charleston, SC, USA

**Keywords:** nurse, suicide, mortality, methods, means, mechanisms

## Abstract

**Aim:**

Examine methods of suicide among nurses cross-nationally.

**Methods:**

The literature was searched to identify epidemiological and cohort studies that analyzed suicide mortality among nurses cross-nationally. Studies were included if nurse suicide mortality was analyzed, and if methods of suicide among nurses were concurrently examined. In total, 22 studies were included, 16 of which were epidemiological and 6 of which were cohort.

**Results:**

Across all studies, nurse suicide decedents from 11 countries were represented. Across most global studies, self-poisoning and hanging were two frequently utilized methods of suicide among nurses. However, in the United States, two common suicide methods included self-poisoning and firearms. While there was likely overlap with respect to public reporting of global cases, in China and India, leading methods included jumping from a building and hanging, respectively. Taken together, despite some inconsistencies, self-poisoning was one of the most frequently reported suicide methods among nurses across studies.

**Conclusion:**

Additional research is important in building the evidence base, particularly with respect to ranking methods of suicide, and further differentiating between suicide methods used by female and male nurses cross-nationally. Additional cross-national research regarding specific substances utilized in suicide self-poisoning deaths is also needed. Because means restriction represents a key suicide prevention strategy, these data are needed to inform means restriction interventions among nurses.

## Introduction

Nurses and midwives comprise half of the global health workforce (World Health Organization [WHO], 2022). The WHO (2022) emphasizes that nurses are essential in delivering high-quality health care across settings, promoting health, and preventing disease. The WHO (2022) further underscores that well-supported nurses are essential in achieving health equity globally. Importantly, the WHO (2022) states that investing in nurses and midwives yields high returns, with respect to improving health, health security, and economic security.

Despite the importance of nurses globally, nurses have encountered numerous challenges in recent years, particularly with respect to the global pandemic. International systematic review evidence suggests nurses and midwives experience elevated rates of suicide when compared to individuals in select occupations and when compared to the general population (Groves et al., 2023b). Regarding surveillance of suicide among nurses, the WHO (2024) reports age-standardized suicide rates broadly; however, occupation-specific suicide rates are not included in these estimates. Methods of suicide are also not included in WHO estimates. Therefore, it is challenging to determine occupation-specific suicide risk, as there is limited availability of data globally. It is also difficult to ascertain the leading methods of suicide among occupational groups cross-nationally.

Therefore, the current review aims to examine methods of suicide among nurses cross-nationally. Reducing access to lethal means represents one of the most internationally efficacious suicide prevention interventions (Substance Abuse and Mental Health Services Administration [SAMHSA], 2017; U.S. Department of Health and Human Services [HHS], 2024). Restricting access to lethal means reduces suicide resulting from those means and may also reduce overall suicide rates (WHO, 2021a). Therefore, taken together, examining methods of suicide among nurses cross-nationally is timely and relevant.

## Methods

The WHO (2024) uses epidemiological studies to guide suicide estimation processes and surveillance of suicide incidence, which is important in countries without or with limited death registration data. Consistent use of epidemiological data increases standardization and allows cross-national comparisons (WHO, 2024). Thus, CINAHL, PsycINFO, PubMed, and Google Scholar were searched to identify epidemiological and cohort studies that simultaneously analyzed both suicide mortality and methods of suicide among nurses. A range of MeSH terms were used to identify nurses broadly across specialties (e.g., licensed nurse, registered nurse, advanced practice nurse). Complementary MeSH terms were also used to identify nurses broadly (e.g., healthcare worker, healthcare provider). MeSH terms related to suicide and methods of suicide were also searched (e.g., intentional self-harm, means).

The literature was searched as part of a wider inquiry, with evidence guiding a series of independent and distinct examinations, including (a) distribution of epidemiological and cohort evidence on nurse suicide mortality globally, quality of global evidence, and global surveillance timeline gaps (Kreuze et al., 2025); (b) epidemiology of suicide among nurses globally, with epidemiological-related gaps in global evidence and cross-regional considerations (Kreuze et al., 2024a); and (c) suicide mortality among nurses in the United States, which integrated mixed methods evidence to examine concurrent issues such as factors associated with suicide and circumstances preceding death (Kreuze et al., 2024b).

All years were actively searched without limits. Epidemiological and cohort studies were included if nurse suicide mortality was analyzed and if methods of suicide among nurses were concurrently examined. While the terms “gender” and “sex” were used interchangeably and/or inconsistently across studies, nurses of all “genders” and “sexes” were eligible for inclusion, and results are reported in terms of gender/sex categories utlized in the studies. Studies that examined nurses broadly were included, as definitions of nurses varied across studies (e.g., licensed nurse, registered nurse, advanced practice nurse). Further, some studies concurrently examined student nurses and midwives, and these studies were also included because the nurse group remained the central focus. In keeping with WHO (2024) surveillance strategies, articles were excluded if they employed a cross-sectional, nonexperimental correlational, or mixed-methods study design. Articles in a non-English language were also excluded.

While searching the literature, five international reviews that examined nurse and/or nursing student suicide were identified. Each review’s reference list was searched to identify applicable studies. The supplementary files of these reviews were also analyzed to identify relevant studies. Examination of the reviews’ reference lists and supplementary files resulted in the inclusion of 20 articles. Subsequent literature searches resulted in the inclusion of two additional articles that were not included in any of these reviews. Together, 22 studies that examined suicide mortality and suicide methods among nurses cross-nationally were included.

## Results

Fourteen epidemiological studies examined methods of suicide among nurses from the following countries (see Table 1): United States (Choflet et al., 2021; Davidson et al., 2018, 2019, 2020), Australia (Kolves & De Leo, 2013; Milner et al., 2016; Petrie et al., 2023), United Kingdom (Hawton et al., 2002; Kelly et al., 1995; National Confidential Inquiry into Suicide and Safety in Mental Health [NCISH], 2020), China (Zeng et al., 2018), Denmark (Hawton et al., 2011), Germany (Peschel et al., 1995), and New Zealand (Skegg et al., 2010). Further, two global epidemiological studies examined methods of suicide among nurses from several countries (Jahan et al., 2021; Rahman & Plummer, 2020). Six cohort studies examined methods of suicide among nurses from the following countries (see Table 1): United States (Davis et al., 2021; Kawachi et al., 1996; Tsai et al., 2015; Wei & Mukamal, 2019), Iceland (Gunnarsdottir & Rafnsson, 1995), and Switzerland (Guseva Canu et al., 2019).

**Table 1.** Methods of Suicide Among Nurses Cross-Nationally.

	Self-poisoning	Hanging, strangulation, and suffocation	Firearms and explosives	Jumping from a height	Drowning	Gas inhalation	Cutting, piercing, and stabbing	Transport vehicle	“Other” methods	Unknown or not recorded
Australia										
Kolves and De Leo (2013)										
Females and males	Nurses more likely to use self-poisoning than referent groups (only method reported)									
Milner et al. (2016)										
Females and males	First leading method	Second leading method	Sixth leading method			Third leading method			Fourth leading method	Fifth leading method
Petrie et al. (2023)										
Females and males	First leading method	Second leading method	Fifth leading method			Fourth leading method			Third leading method	
China										
Zeng et al. (2018)										
Females	Second leading method	Fourth leading method		First leading method		Third leading method	Fifth leading method			
Male				First leading method						
Denmark										
Hawton et al. (2011)										
Females and males	First leading method	Second leading method	Eighth leading method	Fourth leading method	Third leading method	Fifth leading method	Seventh leading method		Sixth leading method	
Females	First leading method	Second leading method	Eighth leading method	Fourth leading method	Third leading method	Fifth leading method	Seventh leading method		Sixth leading method	
Males	Second leading method	First leading method			Tie third leading method	Tie third leading method			Tie third leading method	
Germany										
Peschel et al. (1995)										
Females	Examined self-poisoning substances only									
Male	Examined self-poisoning substances only									
Global samples										
Jahan et al. (2021)										
Females		Second leading method		Tie third leading method	Tie third leading method					First leading method
Male	First leading method									
Rahman and Plummer (2020)										
Females		Tie second leading method			Tie second leading method					First leading method
Male	First leading method									
Iceland										
Gunnarsdottir and Rafnsson (1995)										
Females	First leading method									
New Zealand										
Skegg et al. (2010)										
Females and males	First leading method	Fourth leading method	Fifth leading method			Third leading method			Second leading method	
Switzerland										
Guseva Canu et al. (2019)										
Females	49% died by self-poisoning (only method reported)									
Males	40% died by self-poisoning (only method reported)									
United Kingdom										
Hawton et al. (2002)										
Females	First leading method								Second leading method	
Kelly et al. (1995)										
Females	First leading method	Fourth leading method		Sixth leading method	Fifth leading method	Third leading method			Second leading method	
NCISH (2020)										
Females	First leading method	Second leading method	Tie sixth leading method	Third leading method	Fifth leading method	Tie sixth leading method	Fourth leading method			
Males	22% died by self-poisoning (only method reported)									
United States										
Choflet et al. (2021)										
Females and males	Examined self-poisoning substances only									
Davidson et al. (2018)										
Females and males	First leading method	Third leading method	Second leading method							
Davidson et al. (2019)										
Females and males	First leading method	Third leading method	Second leading method	Fifth leading method	Sixth leading method		Fourth leading method			
Davidson et al. (2020)										
Females	First leading method	Fourth leading method	Second leading method	Tie sixth leading method	Tie sixth leading method		Fifth leading method	Seventh leading method		Third leading method
Males	Second leading method	Fourth leading method	First leading method				Fifth leading method			Third leading method
Davis et al. (2021)										
Females and males	Nurses more likely to use self-poisoning than the general population		Nurses less likely to use a firearms than the general population							
Kawachi et al. (1996)										
Females	First leading method	Second leading method	Fourth leading method		Sixth leading method	Third leading method			Fifth leading method	
Tsai et al. (2015)										
Females	First leading method	Third leading method	Second leading method							
Wei and Mukamal (2019)										
Females	First leading method (only method reported)									

There was considerable variability across studies and cumulative evidence synthesis was somewhat challenging. More specifically, there was a wide range of study periods, different statistical methods (e.g., frequencies by method of suicide, percentages by method of suicide, suicide standardized mortality ratios by method of suicide, the likelihood of method of suicide), variable samples (e.g., female-only samples, female and male samples with data reported for females only), and variability in reporting (e.g., the frequency for one method of suicide only, frequencies for select methods of suicide only, frequencies for leading methods of suicide only, comparison of methods of suicide to a referent group with no frequencies, female-only methods of suicide, sex-differentiated methods of suicide, non-sex-differentiated methods of suicide). Epidemiological and cohort studies were organized according to country, in part, due to the variability across studies. Further, studies were organized according to country in keeping with WHO (2021a) surveillance and prevention strategies.

### Australia

An epidemiological study by Kolves and De Leo (2013) included female and male nurses and had a study period from 1990 to 2007. Female and male nurses were significantly more likely to use self-poisoning when compared to education professionals (44.1% vs. 23.5%) and the general population (44.1% vs. 18.8%). No other nursing-specific suicide method data were provided.

An epidemiological study by Milner et al. (2016) included female and male nurses and had a study period of 2001 to 2012. Female and male nurses and midwives were most likely to use self-poisoning (40%), followed by hanging (28%), carbon monoxide, other methods, not recorded, and firearms. Detailed percentages were provided for self-poisoning and hanging. However, specific values for the remaining methods were not included.

An epidemiological study by Petrie et al. (2023) included female and male nurses and had a study period from 2001 to 2017. Among female and male nurses and midwives, methods included: 145 (45.2%) self-poisoning by chemicals or other substances; 97 (30.2%) hanging, strangulation, and suffocation; 42 (13.1%) other specified means (e.g., drowning, explosive materials, smoke/fire, hot vapors, sharp object, jumping, crashing vehicle); 32 (9.9%) poisoning by gases; <10 (1.6%) firearms; and <10 (0%) other unspecified means.

### China

An epidemiological study by Zeng et al. (2018) included female and male nurses and had a study period from 2007 to 2016. With respect to media and local reporting of nurse suicide in China, 100% of articles reported the suicide method. Among female nurses and nursing students, methods used were: 26 jumping from a building, 4 carbon monoxide poisoning, 3 injection of anesthetic medication, 3 hanging, 2 sleeping pills, 2 poisoning by gas, 2 wrist cutting, 1 injection of potassium cyanide, and 1 injection of pesticide. There was one suicide death in a male nursing student, and the method used for suicide was jumping from a building.

### Denmark

An epidemiological study by Hawton et al. (2011) included female and male nurses and had a study period from 1981 to 2006. Nurses were 4.36 and 3.83 times more likely to use medicinal drugs in fatal overdoses when compared to teachers and the general population, respectively. Among female and male nurses, methods used were: 182 (55%) medicinal drugs, 56 (16.9%) hanging, 38 (11.5%) drowning, 18 (5.4%) jumping, 14 (4.2%) gases and vapors, 12 (3.6%) other methods, 10 (3%) cutting and piercing, and 1 (0.3%) firearms and explosives. Regarding female nurses, methods used included: 177 (56.5%) medicinal drugs, 46 (14.7%) hanging, 37 (11.8%) drowning, 18 (5.8%) jumping, 13 (4.2%) gases and vapors, 11 (3.5%) other methods, 10 (3.2%) cutting and piercing, and 1 (0.3%) firearms and explosives. Regarding male nurses, methods used were: 10 (55.6%) hanging, 5 (27.8%) medicinal drugs, 1 (5.6%) drowning, 1 (5.6%) gases and vapors, and 1 (5.6%) other methods. Female nurses were significantly more likely to use medicinal drugs in fatal suicide overdoses than male nurses.

### Germany

An epidemiological study by Peschel et al. (1995) included female and male nurses and had a study period of 1976 to 1994. Methods used by female nurses included: 2 insulin; 2 injected toxic agent(s), although data on agent(s) were unavailable; 1 polamidon, pethidine, and pentazocine; and 1 thiopental and flunitrazepam. The method used by the male nurse was methohexital and insulin.

### Global Samples

An epidemiological study by Jahan et al. (2021) included female and male nurses, although the year(s) for the study period were not reported. With respect to global press media reporting of nurse suicide, 55.6% of articles reported the suicide method. Among female nurses, methods used were: 4 not reported (Italy, Mexico, Pakistan, United Kingdom); 2 hanging (both from India); 1 jumped from a building (India); and 1 jumped into a river and drowned (Italy). For the male nurse, the method used was opioid overdose (United States).

An epidemiological study by Rahman and Plummer (2020) included female and male nurses and had a study period from March 2020 to June 2020. Regarding global media reporting of nurse suicide, 50% of articles reported the suicide method. Among female nurses, methods used included: 3 not reported (England, Italy, Mexico); 1 jumped into a river (Italy); and 1 hanging (India). For the male nurse, the method used was overdose (United States).

### Iceland

A cohort study by Gunnarsdottir and Rafnsson (1995) included female nurses only and had a study period from 1920 to 1979. Of the five total female nurse suicide deaths, 100% were classified as “suicide and self-inflicted poisoning by analgesic, soporific, or other solid or liquid substances” (p. 27). The standardized mortality ratio (SMR) for self-poisoning among female nurses was 4.59 (i.e., there were more self-poisoning suicides among nurses than would be expected). Of the five female suicides, 100% were among nurses employed less than 20 years. The SMR for self-poisoning among female nurses employed less than 20 years was 11.11 (i.e., there were more self-poisoning suicides among nurses employed less than 20 years than would be expected).

### New Zealand

An epidemiological study by Skegg et al. (2010) included female and male nurses and had a study period from 1973 to 2004, although nurse-specific suicide data were unavailable between 1996 and 1997. Among female and male nurses, suicide methods included: 47 (44.3%) poisoning, 20 (18.9%) other, 17 (16%) motor vehicle exhaust and other gases, 16 (15.1%) hanging, and 6 (5.7%) firearms.

### Switzerland

A cohort study by Guseva Canu et al. (2019) included female and male nurses and had a study period from 1990 to 2014. Data on one suicide method among nurses was examined (i.e., self-poisoning). Among female nurses, 49% died by self-poisoning. Among male nurses, 40% died by self-poisoning. Data on other methods of suicide were not provided.

### United Kingdom

An epidemiological study by Hawton et al. (2002) included female nurses only and had a study period from 1994 to 1997. Among female nurses, the methods used were: 72 (67.9%) self-poisoning, 32 (30.2%) self-injury, and 2 (1.9%) used both self-poisoning and self-injury. Hanging comprised many self-injury cases (18 deaths). Drugs used for self-poisoning included: 28 antidepressants, 21 nonopiate analgesics, 12 opiates, 3 insulin, and 1 anesthetic agent. Of the nonopiate analgesics, 16 (76.2%) included co-proxamol or paracetamol with dextropropoxyphene. Among the four total cases that included insulin and an anesthetic agent, medications were taken from the nurse decedents’ workplace. When methods used for suicide among female nurses were compared to methods used for suicide among similarly aged adult females in England and Wales, self-poisoning using drugs was modestly more common among female nurses (57.6% vs. 49.1%).

An epidemiological study by Kelly et al. (1995) included females and males, although suicide data were available for female nurses only. The study period was 1982 to 1992. Suicide methods among female nurses included: 58% poisoning, 13% other, 10% car gas, 8% hanging, 6% drowning, and 5% jumping from a height. With respect to self-poisoning, 33.3% of female nurses used analgesics, antipyretics, and antirheumatics (which included paracetamol). Further, tranquillizers were used by 20% of female nurses, and psychotropic drugs (which included antidepressants) were used by 30% of female nurses.

An epidemiological study by the NCISH (2020) included female and male nurses and had a study period from 2011 to 2016. Among female nurses, suicide methods included: 85 (42%) self-poisoning, 80 (39%) hanging/strangulation, 10 (5%) jumping/multiple injuries, 7 (4%) cutting/stabbing, 6 (3%) drowning, <3 gas inhalation, and <3 firearms. Female nurses were significantly more likely than females in other occupations to die by self-poisoning (42% vs. 30%). With respect to mental health co-morbidities, female nurses in contact with mental health services in the year preceding death used the following methods: 39 (48%) self-poisoning, 28 (35%) hanging/strangulation, 4 (5%) cutting/stabbing, <3 jumping/multiple injuries, <3 drowning, <3 gas inhalation, and <3 firearms. Regarding self-poisoning, female nurses in contact with mental health services used: psychotropics (33%), opiates (31%), and paracetamol (19%). Female nurses in contact with mental health services were significantly more likely to die by self-poisoning than females in other occupations in contact with mental health services (48% vs. 30%). More specifically, female nurses in contact with mental health services were significantly more likely to use paracetamol than females in other occupations in contact with mental health services (19% vs. 10%). Data on suicide among male nurses was limited. Male nurses were more likely to die by self-poisoning than males in other occupations (22% vs. 12%). However, fewer male nurses died by self-poisoning than female nurses (22% vs. 42%).

### United States

An epidemiological study by Choflet et al. (2021) included female and male nurses and had a study period from 2003 to 2017. Among female and male nurses, the following substances were listed as a contributing cause of death: 358 (44.64%) opioid, 353 (44.01%) antidepressant, 350 (43.64%) benzodiazepine, 338 (42.14%) alcohol, 131 (16.33%) antihistamine, 125 (15.59%) miscellaneous, 95 (11.85%) substances of abuse, 70 (8.73%) stimulant, 67 (8.35%) anticonvulsant, 58 (7.23%) acetaminophen, 58 (7.23%) caffeine, 56 (6.98%) antipsychotic, 43 (5.36%) inhalant, 39 (4.86%) barbiturate, 39 (4.86%) muscle relaxant, 38 (4.74%) nonbenzodiazepine sedative, 28 (3.49%) tetrahydrocannabinol, 24 (2.99%) nicotine, 21 (2.62%) diverted substances, and 21 (2.62%) poison. When nurses were compared to non-nurses, 19 substances were significantly more likely to be listed as a contributing cause of death among nurses, with the top five being: opioid (44.64% vs. 11.81%), antidepressant (44.01% vs. 9.4%), benzodiazepine (43.64% vs. 10.99%), alcohol (42.14% vs. 13.01%), and antihistamine (16.33% vs. 5.5%). However, importantly, the authors note that it is unclear if these substances represent the sole cause of death or if they represent factors that contributed to death. Further, the authors note that more than one substance may be used in a single suicide case, although there was no co-substance use data among nurse suicide decedents.

An epidemiological study by Davidson et al. (2018) included female and male nurses and had a study period from 2005 to 2015. Among female and male nurses, the five most frequently utilized methods were: 26 (44.83%) drugs, 11 (18.97%) firearms, 6 (10.34%) alcohol-drug combinations, 4 (6.9%) hanging, and 3 (5.17%) plastic bag suffocation. Regarding self-poisoning, the 20 most frequently utilized medications included: 10 diphenhydramine, 9 acetaminophen, 7 alcohol, 7 hydrocodone, 5 quetiapine, 5 zolpidem, 4 citalopram, 4 oxycodone, 3 alprazolam, 3 bupropion, 3 clonazepam, 3 venlafaxine, 2 amitriptyline, 2 benzodiazepines, 2 diltiazem, 2 lorazepam, 2 propoxyphene, 2 temazepam, 1 amlodipine, and 1 aspirin.

An epidemiological study by Davidson et al. (2019) included female and male nurses and the study period included 2014. Among female and male nurses, methods included: 72 (35.1%) pharmaceutical poisoning; 69 (33.7%) firearms; 28 (13.7%) hanging, strangulation, and suffocation; 26 (12.7%) other poisoning; 5 (2.4%) sharp instrument; 4 (2%) fall; and 1 (0.5%) drowning. With respect to self-poisoning, the following substances were listed as a contributing cause of death: benzodiazepines (2 alprazolam, 1 diazepam, 1 lorazepam, 1 benzodiazepines); opioids (2 opiates, 2 tramadol, 1 oxycodone, 1 morphine, 1 hydrocodone); tricyclic antidepressants (2 amitriptyline, 2 cyclobenzaprine); antihistamines (3 promethazine, 1 cetirizine, 1 hydroxyzine); anticonvulsants (2 gabapentin, 1 lamotrigine); Selective Serotonin Reuptake Inhibitor (1 citalopram, 1 fluoxetine, 1 paroxetine); antipsychotic/D2 antagonist (1 haloperidol); barbiturates (1 phenobarbital); nonbenzodiazepine sedative-hypnotics (2 trazodone, 1 zolpidem); and miscellaneous (1 potassium, 1 succinylcholine, 1 verapamil, 1 ibuprofen, 1 insulin, 1 dextromethorphan, 1 bupropion, 1 buspirone).

An epidemiological study by Davidson et al. (2020) included female and male nurses and had a study period from 2005 to 2016. Among female nurses, methods were: 399 (27.2%) pharmacologic poisoning; 333 (22.7%) firearms; 254 (17.3%) other poisoning; 219 (14.9%) unknown; 183 (12.5%) hanging, strangulation, and suffocation; 32 (2.2%) sharp instrument; 16 (1.1%) fall; 16 (1.1%) drowning; and 5 (0.3%) other transport vehicle. Among male nurses, methods included: 148 (41.7%) firearms; 74 (20.8%) unknown; 43 (12.1%) hanging, strangulation, and suffocation; 43 (12.1%) other poisoning; 32 (9%) pharmacologic poisoning; and 8 (2.3%) sharp instrument. Male nurses were significantly more likely to use firearms than female nurses. Regarding pharmacologic self-poisoning among female and male nurses, the most frequently used substances were: 476 other, 425 opiate, 354 antidepressant, 271 benzodiazepines, 94 alcohol, 64 antipsychotic, 62 muscle relaxant, 46 anticonvulsant, and 20 barbiturates. Within the “other” category, the following medications were listed as a contributing cause of death: 56 acetaminophen, 36 cyclobenzaprine, 18 metoprolol, 13 insulin, 12 propanolol, 11 propofol, 9 humulin, 9 propoxyphene hydrochloride, 9 verapamil, 8 salicylates, 7 carisoprodol, 6 diltiazem, 6 soma, 5 buspirone, 5 flecainide, and 5 meprobamate. Within the “drugs of abuse” category, the following were listed as a contributing cause of death: 18 amphetamine and 10 cocaine.

A cohort study by Davis et al. (2021) included female and male nurses and had a study period from 2007 to 2018. Female and male nurses were statistically less likely to use a firearm when compared to suicide decedents from the general population, although specific percentages were not reported. Female and male nurses were statistically more likely to use self-poisoning when compared to suicide decedents from the general population (24.9% vs. 16.8%). When nurse suicide decedents were compared to suicide decedents from the general population, the following substances were significantly more likely to be used by nurses: antidepressants (44% vs. 36%), benzodiazepines (42% vs. 32.7%), and opiates (33.7% vs. 27.4%).

A cohort study by Kawachi et al. (1996) included female nurses only and had a study period from 1980 to 1990. Methods used by female nurses included: 21(37.5%) medication overdose, 10 (17.9%) hanging, 9 (16%) poisoning by gas, 8 (14.3%) firearms, 5 (8.9%) miscellaneous, and 3 (5.4%) drowning.

A cohort study by Tsai et al. (2015) included female nurses only and had a study period from 1992 to 2010. The most frequently utilized methods among female nurses were: 21 (48.8%) poisoning by solid or liquid substances, 8 (18.6%) firearms and explosives, and 6 (14%) strangulation and suffocation. Data on the remaining methods were not provided.

A cohort study by Wei and Mukamal (2019) included female nurses only and had study periods from 1992 to 2016 and 1993 to 2016. The most common method of suicide among female nurses was “poisoning by solid or liquid substances” (p. 513). No other data on suicide methods were provided.

### Summary

While the WHO (2024) reports age-standardized suicide rates, occupation-specific suicide rates are not included in these estimates. Similarly, methods of suicide are also not included in WHO estimates. Because it is difficult ascertaining leading methods of suicide among occupational groups cross-nationally, the literature was searched to identify methods of suicide among nurses.

Cumulatively, 27.3% of studies explored only one suicide method and/or did not provide rankings or detailed percentages for suicide methods among nurses, making it difficult to ascertain leading methods. Further, while self-poisoning was frequently cited as the leading method of suicide across studies, only 50% of studies included specific descriptions regarding substances implicated in suicide self-poisoning deaths. Across all studies, 36.4% differentiated between female and male suicide methods, 36.4% presented results for combined female and male suicide methods only with no differentiation between sexes, 22.7% included female-only samples and presented suicide methods for females only, and 4.5% included a female and male sample although nurse suicide data were available for females only. Taken together, additional cross-national research on methods of suicide among nurses is important in building the evidence base. Further, broader research is also important, as there is limited referent data regarding methods of suicide by occupation.

Across all studies, there were differentiated data for female and male suicide methods for the following countries: China, Denmark, Germany, Switzerland, the United Kingdom, and the United States. However, there were no sex-specific suicide method data for Australia and New Zealand, as females and males were combined. Further, suicide methods were available among females only for Iceland. Additional research is important in building the evidence base, particularly with respect to ranking methods of suicide and further differentiating between suicide methods used by female and male nurses cross-nationally. Additional research regarding specific substances utilized in suicide self-poisoning deaths is also needed, as most data were derived from the United States and the United Kingdom, and there was limited differentiation of data by sex.

Across studies, nurses from nine countries were represented. However, when also including studies with global samples, nurse suicide decedents from 11 total countries were represented. Expanded research is important across additional countries, as more than half of studies included nurse suicide decedents from the United States (36.4%), Australia (13.6%), and the United Kingdom (13.6%). Taken together, it remains difficult to ascertain frequently utilized suicide methods among nurses, due to the limited availability of cross-national data. Additional epidemiological and cohort research is needed to build the evidence base and to facilitate comparisons regarding methods of suicide among nurses.

## Discussion

Because nurses comprise a substantial proportion of the global health workforce (WHO, 2022), cross-national surveillance regarding the incidence of suicide and methods of suicide among nurses is important. An essential WHO (2023) sustainable development goal includes enhancing the availability of timely data and estimates that may be used to guide policy and action across multiple levels. As part of this cross-national surveillance, identifying leading methods of suicide among female and male nurses is important. Data on methods of suicide are especially important in informing prevention initiatives. Reducing access to lethal means represents a key universal suicide prevention strategy (HHS, 2024; SAMHSA, 2017). Restricting access to lethal means reduces suicide resulting from those means and may also reduce overall suicide rates (WHO, 2021a). Therefore, data on methods of suicide are needed to inform progress toward the concurrent WHO (2021b) sustainable development goal of reducing suicide mortality.

More specifically, identifying the leading methods of suicide among nurses cross-nationally is significant, as reducing access to lethal means represents one of the most internationally efficacious suicide prevention interventions (HHS, 2024; SAMHSA, 2017; WHO, 2021a). When access to lethal means is restricted, it provides critical time for individuals experiencing acute distress to receive support, allowing crises to pass before individuals take fatal action (WHO, 2021a). To develop efficacious cross-national suicide prevention interventions, understanding commonly utilized methods of suicide among nurses in each country is critical, in part, because methods of suicide vary geographically (e.g., low-income vs. high-income country) and also vary by sociodemographic characteristics (e.g., sex, age; WHO, 2021a). Taken together, additional cross-national research is important in identifying the most commonly utilized methods of suicide among nurses. This research must also be sustained, as methods of suicide may vary with time (WHO, 2021a). Collectively, these data are needed to inform ongoing prevention with respect to preventing suicide among nurses.

Complementary concurrent policy research at the organizational and national levels may also be important, particularly with respect to reducing access to lethal means (e.g., reducing nurses’ access to lethal substances in the workplace). Engaging both private (e.g., workplaces) and public (e.g., government, legislators) sectors may improve coordination and increase both the reach and impact of suicide prevention initiatives (HHS, 2024; WHO, 2021a). Consequently, the WHO (2021a) endorses a collaborative, multisectoral approach between diverse stakeholders.

Additional cross-sector, cross-national research is important, as the single systematic review on suicide among nurses and midwives identified only one suicide prevention intervention for nurses, which was based in the United States (Groves et al., 2023b). Somewhat similarly, the single systematic review on suicide among nursing and midwifery students identified only three suicide prevention interventions for students, one of which was based in the United States, one of which was based in Canada, and one of which was based in India (Groves et al., 2023a). Identifying leading methods of suicide among nurses cross-nationally may help to inform the development of additional suicide prevention interventions that reduce access to lethal means, which could thereby reduce nurse suicide mortality.

## Conclusion

Because it is difficult to ascertain leading methods of suicide among occupational groups cross-nationally, the literature was searched to identify methods of suicide among nurses. Taken together, additional research is important in building the evidence base, particularly with respect to ranking methods of suicide, identifying specific substances implicated in self-poisoning deaths, and further differentiating methods of suicide by sex and country. Because reducing access to lethal means represents a key universal suicide prevention strategy, it is important to build the evidence base regarding methods of suicide among nurses, as these data are needed to inform the development of suicide prevention interventions that reduce access to these lethal means.
